# Draft Genome Sequence of Salinisphaera sp. Strain KSM-18, an Obligately Halophilic Bacterium Isolated from a Triassic Salt Mine

**DOI:** 10.1128/MRA.00897-18

**Published:** 2018-08-16

**Authors:** Stephen A. Kelly, Julianne Megaw, Brendan F. Gilmore

**Affiliations:** a School of Pharmacy, Queen’s University Belfast, Belfast, United Kingdom; Louisiana State University

## Abstract

Here, we report the draft genome sequence of Salinisphaera sp. strain KSM-18. This obligately halophilic bacterium was isolated from a brine sample obtained from a Triassic salt mine.

## Abstract (precis)

Here, we report the draft genome sequence of Salinisphaera sp. strain KSM-18.

## ANNOUNCEMENT

The genus Salinisphaera is member of the class *Gammaproteobacteria*, which contains several medically important species of bacteria, such as Escherichia coli, Pseudomonas aeruginosa, and Vibrio cholerae, among others. Salinisphaera spp. contain Gram-negative, short rod-shaped or coccoid bacteria, first described in 2003 after the isolation of the new genus and species Salinisphaera shabanensis from the brine-seawater interface of the Shaban Deep in the northern Red Sea (1). Representatives of this genus have since been isolated from diverse saline environments, such as deep-sea hydrothermal vents, surface seawater, solar salterns, brine from a salt well, and the surface of a deep-sea fish. Salinisphaera species have been described as both halotolerant and moderate halophiles, capable of growth in NaCl concentrations from 1 to 30% (wt/vol) (2–6).

Strain KSM-18 was isolated from a sample of brine collected from the Kilroot salt mine, a Triassic halite deposit located in Carrickfergus, Northern Ireland, onto agar containing (per liter) yeast extract (10 g), casein hydrolysate (7.5 g), glycerol (10 ml), agar (15 g), and 25% rock salt from the Kilroot salt mine at 37°C. The strain showed no growth in the absence of salt and could be recovered only over the salinity range of 20 to 25% NaCl. Based on 16S rRNA gene sequence similarity, the organism was found to be a member of the genus Salinisphaera, with the closest neighbor deemed to be Salinisphaera halophila strain USBA_874 (99% similarity) (7). The genome of strain KSM-18 is worth investigating and mining for functional genes, such as those encoding antimicrobials or biocatalysts, especially given the ancient, extreme, and relatively undisturbed nature of the organism’s source environment. This has led to the environment possessing a unique microbiome, which may provide clues to the evolutionary path taken by ancient microbes.

Genomic DNA was extracted from a subculture of strain KSM-18 using a GenElute bacterial genomic DNA kit (Sigma-Aldrich, United Kingdom), following the protocol for Gram-negative bacteria, yielding 8.92 μg DNA. Whole-genome sequencing was performed by MR DNA (Shallowater, TX), using the Illumina MiSeq platform (40× coverage). The sequence reads were assembled *de novo* by MR DNA using the NGen DNA assembly software by DNAStar, Inc. The assembled genome contained 5 contigs (the longest was 1,614,654 bp) with a total size of 2,710,038 bp and a GC content of 59.6%. Annotation in Rapid Annotations using Subsystems Technology (RAST) (8) revealed 181 subsystems, 2,740 coding sequences, and 50 RNAs. Several genes were identified that were associated with resistance to heavy metals and toxic compounds (arsenic, cobalt, copper, mercury, and zinc), antibiotic resistance (fluoroquinolones, β-lactams, and multidrug resistance genes), and genes for several classes of potentially biocatalytic enzymes (transaminases, monooxygenases, esterases, and lipases).

### Data availability.

This whole-genome shotgun project has been deposited at DDBJ/ENA/GenBank under the accession number QJOD00000000. The version described in this paper is version QJOD01000000.
